# Proteomic Analysis of Kveim Reagent Identifies Targets of Cellular Immunity in Sarcoidosis

**DOI:** 10.1371/journal.pone.0170285

**Published:** 2017-01-23

**Authors:** Christian Eberhardt, Muhunthan Thillai, Robert Parker, Nazneen Siddiqui, Lee Potiphar, Rob Goldin, John F. Timms, Athol U. Wells, Onn M. Kon, Melissa Wickremasinghe, Donald Mitchell, Mark E. Weeks, Ajit Lalvani

**Affiliations:** 1 Tuberculosis Research Unit, National Heart and Lung Institute, Imperial College London, London, United Kingdom; 2 Cambridge Interstitial Lung Disease Group, Papworth NHS Trust, Cambridge, United Kingdom; 3 Centre for Pathology, Department of Medicine, Imperial College London, London, United Kingdom; 4 Institute for Women's Health, Faculty of Population Health Sciences, University College London, London, United Kingdom; 5 Interstitial Lung Unit, Royal Brompton Hospital, Imperial College London NHS Healthcare Trust, London, United Kingdom; 6 Department of Respiratory Medicine, St. Mary’s Hospital London, Imperial College London NHS Healthcare Trust, London, United Kingdom; 7 Barts Cancer Institute- a Cancer Research UK Centre of Excellence, Queen Mary University of London, John Vane Science Centre, Charterhouse Square, London EC1M 6BQ, United Kingdom; Universita degli Studi di Palermo, ITALY

## Abstract

**Background:**

Kveim-reagent (Kv) skin testing was a historical method of diagnosing sarcoidosis. Intradermal injection of treated sarcoidosis spleen tissue resulted in a granuloma response at injection site by 4–6 weeks. Previous work indicates proteins as the possible trigger of this reaction. We aimed to identify Kv-specific proteins and characterise the *ex vivo* response of Peripheral Blood Mononuclear Cells (PBMCs) from sarcoidosis, tuberculosis and healthy control patients when stimulated with both Kv and selected Kv-specific proteins.

**Methods:**

Kv extracts were separated by 1D-SDS-PAGE and 2D-DIGE and then underwent mass spectrometric analysis for protein identification. Sarcoidosis and control PBMCs were first stimulated with Kv and then with three selected recombinant protein candidates which were identified from the proteomic analysis. PBMC secreted cytokines were subsequently measured by Multiplex Cytokine Assay.

**Results:**

We observed significantly increased IFN-γ and TNF-α secretion from Kv-stimulated PBMCs of sarcoidosis patients vs. PBMCs from healthy volunteers (IFN-γ: 207.2 pg/mL vs. 3.86 pg/mL, *p* = 0.0018; TNF-α: 2375 pg/mL vs. 42.82 pg/mL, *p* = 0.0003). Through proteomic approaches we then identified 74 sarcoidosis tissue-specific proteins. Of these, 3 proteins (vimentin, tubulin and alpha-actinin-4) were identified using both 1D-SDS-PAGE and 2D-DIGE. Data are available via ProteomeXchange with identifier PXD005150. Increased cytokine secretion was subsequently observed with vimentin stimulation of sarcoidosis PBMCs vs. tuberculosis PBMCs (IFN-γ: 396.6 pg/mL vs 0.1 pg/mL, *p* = 0.0009; TNF-α: 1139 pg/mL vs 0.1 pg/mL, *p*<0.0001). This finding was also observed in vimentin stimulation of sarcoidosis PBMCs compared to PBMCs from healthy controls (IFN-γ: 396.6 pg/mL vs. 0.1 pg/mL, *p* = 0.014; TNF-α: 1139 pg/mL vs 42.29 pg/mL, *p* = 0.027). No difference was found in cytokine secretion between sarcoidosis and control PBMCs when stimulated with either tubulin or alpha-actinin-4.

**Conclusions:**

Stimulation with both Kveim reagent and vimentin induces a specific pro-inflammatory cytokine secretion from sarcoidosis PBMCs. Further investigation of cellular immune responses to Kveim-specific proteins may identify novel biomarkers to assist the diagnosis of sarcoidosis.

## Introduction

Sarcoidosis is a multi-organ granulomatous disease of unknown cause which occurs in genetically susceptible individuals [1] but primarily affects the lungs. The worldwide prevalence is 40 per 100,000 with highest incidence in North America, Scandinavia and Japan [2]. Despite evidence for environmental triggers including clustered outbreaks and person-to-person transmission [3], there is no universally accepted cause of disease. The largest case controlled study to date comprised 705 patients and controls did not identify any common predominant triggers [4].

Diagnosis of sarcoidosis is complex and relies on a supportive clinical history, radiology and biopsy exhibiting non-caseating granulomas. This approach is resource-heavy and merely suggestive of disease through exclusion of differential diagnoses, rather than specifically diagnosing sarcoidosis [5]. Historically an *in vivo* skin assay called the Kveim test, was used for diagnosis with sensitivity >70% and specificity >90% [6]. Kveim reagent (Kv) was a homogenized, heated suspension of sarcoidosis spleen tissue, injected intradermally to produce a pathognomonic reaction at 4–6 weeks [7]. Biopsy of the injection site revealed granulomas identical to that in diseased organs, indicating a shared immune response between the reaction and the disease itself. Kv testing is no longer in clinical use due to the possibility of disease transmission between individuals, discounting the possibility of future *in vivo* human studies.

Despite extensive clinical validation, there has been limited successful research into the triggers of the Kv reaction. A sequential removal of lipids and oligosaccharides did not alter the granuloma-causing capacity of Kv whereas concentration of proteins improved sensitivity, suggesting the cause is likely protein-driven [8]. Immunological analysis of T-cell receptors at the injection site identified an influx of oligoclonal CD4+ T-cells, indicating a limited number of T-cell antigenic targets [9].

One previous proteomic analysis of sarcoidosis solid tissue did identify the mycobacterial protein mKatG within Kv [10]. A further study by the same group demonstrated higher CD4+ T-cell responses towards mKatG in sarcoidosis compared to healthy volunteers with evidence of compartmentalization of response in the lungs of patients, indicating that it may be one of several pathogenic antigen in sarcoidosis [11].

We postulated that early antigen-driven immune responses contributing to the generation of the *in vivo* Kv-induced granuloma at 4–6 weeks would also be detectable *ex vivo* in peripheral blood. We aimed to define the proteomic signature of Kv itself and to characterise the nature of the *ex vivo* immune response to both Kv and selected identified Kv-specific proteins.

## Material and Methods

### Ethics statement

This study was approved by the St. Mary’s institutional ethics committee (reference: 07/H0712/85) and blood was obtained from participants after providing written informed consent. All sarcoidosis tissue was collected under the same ethical agreement.

### Patient recruitment

Sarcoidosis patients were chosen who had recent biopsy-proven pulmonary disease and were not on immunosuppressive therapy; diagnosis was obtained as per ATS guidelines [5]. Tuberculosis patients had culture confirmed disease and were recruited prior to anti-tuberculous therapy. Healthy volunteers were recruited specifically for this study.

### Preparation of Kv and recombinant proteins

Sarcoidosis spleen tissue and control spleen was provided by National Disease Research Interchange (Philadelphia, United States). Validated Kv was provided by Alvin Teirstein and Porton Down Institute. The method for the preparation of Kv follows the original protocol exactly [7]. For PBMC stimulation, 100 μL suspended Kv was precipitated using 2D-clean-up-kit (GE Healthcare, Piscataway, NJ, USA) and the pellet was dissolved under sonication in 600 μL RMPI-1640 (Sigma-Aldrich). Individual identified proteins were purchased as recombinant proteins (Abcam, Cambridge, UK) and dissolved in RMPI-1640 at 20 μg/mL.

### PBMC isolation and antigen stimulation

2.5 x10^5^ PBMCs in a total volume of 100 μL of AIM V media (Sigma-Aldrich) were placed per well in flat bottom plates (Thermo-Fisher). For stimulation, PBMCs were either supplemented with 20 μL of Kv or recombinant protein (20mg/ul of RPMI). Plates were incubated for 36 hrs at 37°C and 5% CO_2_ and spun at 500 g for 1 min at 21°C. Supernatant was aliquoted for immediate analysis.

### Multiplex cytokine analysis

The multiplex cytokine analysis was performed using pre-coated 96 well plates (Human Pro-inflammatory I Ultrasensitive Assay, Meso Scale Discovery–MSD, Maryland, USA) according to the manufacturer instructions and as previously described [12]. 25 μL of the supernatant derived from PBMC incubation with antigen (with PHA ensuring PBMC viability as a positive control) was added per well and samples measured in duplicate. Mean values of duplicate wells were recorded after subtraction of negative control. Plates were analysed using the MSD SECTOR Imager 2400 and Discovery Workbench 3.0 software (Meso Scale Discovery, USA). Statistical significance to look for differences between groups was determined by Mann-Whitney test.

### 1D-SDS-PAGE

Protein extracts of Kv were quantified (Bio-Rad, Hercules, CA) and 4 μg loaded onto 4–20% polyacrylamide gels (Bio-Rad Laboratories, Hercules, California, USA), as reported previously [13]. Gels were stained with colloidal Coomassie-Blue solution and immediately scanned (Seiko-Epson, Nagano, Japan).

### 2D-DIGE

Experiments were conducted as previously outlined [14]. 50 μg of spleen protein extract was labelled with fluorescent dye (N-hydroxysuccinimidyl (NHS) Cy2, Cy3 and Cy5) (GE Healthcare, Piscataway, NJ, USA) and separated by 2D-PAGE. Samples were cup loaded onto IPG strips, underwent first-dimension (1D) isoelectric focusing (IEF) over 16 hours (1D) (Ettan IPGPhor 3, GE Healthcare) and subsequent second-dimension (2D) SDS-PAGE (Ettan DALT 12, GE Healthcare). Preparative gels for protein identification were loaded with 450 μg protein per gel. Gels were run in duplicate at each pI 3–7 and 6–9 and imaged by dye excitation using a Typhoon 9400 scanner (GE Healthcare). Images were analysed using Progenesis SameSpots version 3.0 (Nonlinear Dynamics, Newcastle, UK).

### MS/ MS

Each gel lane of a 1D-SDS-PAGE gel was cut into 10 sections and selected gel spots of the 2D-DIGE gels were also excised with a scalpel. 1D-SDS-PAGE gel slices and 2D-DIGE gel spots underwent subsequent in-gel tryptic digest as follows: in brief, gel pieces were washed in 30 μL 50% acetonitrile (ACN) for protein destaining. Gel pieces were dried in a SpeedVac for 10 min, reduced with 15 μL 10mM DTT (Sigma-Aldrich) in 10 mM ammonium bicarbonate pH 8.0 (AmBic) for 45 min at 50°C and then alkylated with 15 μL 50 mM IAM (Bioultra-Sigma-Aldrich) in 10 mM AmBic for 1 h at room temperature in the dark. Gel pieces were washed three times in 30 μL 50% ACN, vacuum-dried before being reswollen with 50 ng of modified trypsin (Promega, Southampton, UK) in 5 μL 10mM AmBic and eventually overlaid with 10 μL 10mM AmBic. After incubation for 16 h at 37°C peptides were extracted twice with 10 μL 5% TFA (Sigma-Aldrich) in 50% ACN, vacuum-dried, resuspended in 8.0 μL of 0.1% (v/v) formic acid and stored at -80°C until further use.

Mass spectrometric identification was conducted as previously described [15] using electrospray Q-TOF LC-MS/MS with a chip cube interface (model 6520, Agilent Technologies, Santa Clara, California, USA). Essentially, obtained tryptic peptides (7 μL) were loaded onto the enrichment column of the chip and washed with eight column volumes of 0.1% trifluoroacetic acid (TFA). Tryptic peptides were separated on the analytical column using an acetonitrile gradient 4.0% to 50% [v/v]) over 19 min and eluted directly into the mass spectrometer. The mass spectrometer was run in positive ion mode, and MS survey scans were run over a range of *m/z* 250 to 3,000 and at five spectra per second. Precursor ions were selected for automatic tandem MS (MS/MS) at an absolute threshold of 2,000 and a relative threshold of 0.01, with a maximum of five precursors per cycle.

Resulting MS/MS data was analysed using MassHunter Mass Profiler software version B.02.01 (Agilent Technologies). Data were then searched with MASCOT Version 2.2.07 (Matrix Science, London, UK) against UniProtKB/Swiss-Prot Database Release 2011_11 (533049 sequences; 189064225 residues) for the DIGE dataset or against UniProtKB/Swiss-Prot Database Release 2012_01 (534242 sequences; 189454791 residues) for the Kveim dataset. Database search was performed with settings as follows: taxonomy filter was set for human entries, MS tolerance (Parent Tolerance) ±20 ppm and the MS/MS tolerance (Fragment Tolerance) ±0.20 Da. Trypsin was set as the digesting enzyme, two missed cleavages of the digest were accepted and carbamidomethylation of cysteines (C) was set as fixed modification. Acetylation of peptide N-termini and lysine residues (K), methionine oxidation (M), deamidated (NQ) and Gln-pyroGlu (N-term Q) as well as amidated C-termini, carbamylated lysine residues and carbamylated N-termini were selected as variable modifications.

Search result filters were set to MudPIT scoring, permitted peptides with a score >20 and below the Mascot significance threshold filter of p = 0.01 as valid identification. Resulting dat.-files were then imported into Scaffold Version 3.4.7 (DIGE data) or 3.6.7 (Kveim data) (Scaffold, Proteome Software, Portland, OR, USA) and an additional database search with the X! Tandem algorithm (Version CYCLONE 2010.12.01.1) was performed with equally stringend search criteria. Besides aforementioned modification pyro-cmC (S-carbamoylmethylcysteine cyclization) on N-termini was added as another variable modification. Here, data are presented in Scaffold that were confidently identified by the two independent search engines i.e. Mascot and X! Tandem and further fulfil the Scaffold implemented criteria of 99% minimum protein probability score, 95% minimum peptide probability score with at least two identified peptides. The mass spectrometry proteomics data have been deposited to the ProteomeXchange Consortium via the PRIDE [16] partner repository with the dataset identifier PXD005150 and 10.6019/PXD005150.

### Immunohistochemistry (IHC)

Monoclonal mouse anti-Vimentin, (Code No. M 0725, Dako, Carpinteria, CA, USA), was applied as primary antibody at a 1:50 dilution on 8 μm cryosections of snap frozen spleen tissue for 30 min at room temperature. Dako Cytomation Mouse lgG'1, (Code No. X 0931, Dako), diluted to the same mouse lgG concentration as the primary mouse anti-Vimentin antibody was used as negative control and run simultaneously with sarcoid spleen sections. For visualization, DAKO LSAB2 kit (Code No. K 0679) and DAKO EnVision+/HRP kits, (Code Nos. K 4004 and K 4006) were used on the Bond autostainer for automated IHC staining.

### Statistical analysis

For each direct comparison (e.g. measurement of secreted interferon gamma after vimentin stimulation of sarcoidosis PBMCs vs tuberculosis PBMCs) a p-value was calculated using a Mann-Whitney test. Data analysis was performed using GraphPad Prism version 6 for Macintosh (La Jolla, California, USA).

## Results

### Stimulation with Kv induces IFN-γ/ TNF-α secretion from sarcoidosis PBMCs

**Fig 1:**
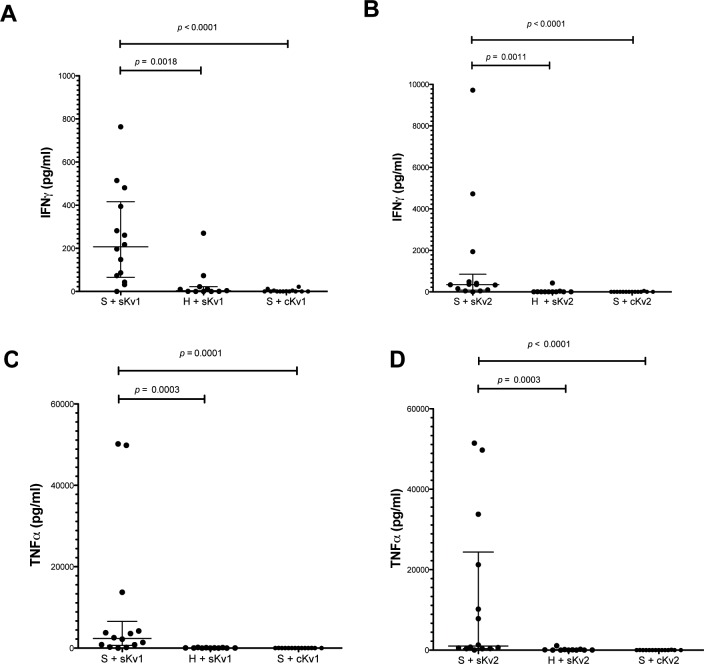
Total IFN-γ (Fig 1a) and TNF-α secretion (Fig 1c) after incubation of PBMCs from patients with confirmed Sarcoidosis (S) or healthy volunteers (H) with sarcoidosis (sKv) or control (cKv) Kveim reagent. Each dot represents mean cytokine concentration of one well stimulated in duplicate with antigen for 36 hrs. Fig 1b and 1d represent replicate experiments with the same PBMCs but using replicates of sKv and cKv. Statistical significance for each comparison was determined by Mann-Whitney test.

PBMCs from sarcoidosis patients and healthy volunteers were stimulated with both sarcoidosis Kv (sKv) and healthy control spleen prepared in a manner similar to Kveim (cKv). The secreted cytokine signature was then analysed. The median IFN-γ concentration was significantly elevated in the cell culture supernatant of PBMCs from patients with active pulmonary sarcoidosis (Table 1) stimulated with sKv (207.2 pg/mL, IQR 65.94–416.1) compared to either healthy control PBMCs stimulated with sKv (3.86 pg/mL, IQR 0.1–22.32, *p* = 0.0018) or sarcoidosis PBMCs stimulated with cKv (0.1 pg/mL IQR 0.1–4.37, *p*<0.0001) as seen in Fig 1A.

**Table 1:** 

KV study population	Pulmonary sarcoidosis	Healthy volunteers	
Number	14	11	
Pulmonary disease only (%)	12 (86)	n/a	
Median age (range)	46.5 (28–71)	33 (23–49)	
Female gender (%)	5 (36)	7 (64)	
Ethnic origin: Caucasian/Asian/Black	7/ 4/ 3	8/ 2/ 1	
Time since diagnosis	0–4 weeks	n/a	
			
Vimentin study population	Pulmonary sarcoidosis	Healthy volunteers	Pulmonary tuberculosis
Number	14	8	15
Pulmonary disease only (%)	11 (79)	n/a	15 (100)
Median age (range)	48.5 (28–71)	35.5 (25–43)	32 (20–54)
Female gender (%)	4 (29)	3 (37.5)	9 (60)
Ethnic origin: Caucasian/Asian/Black	5/ 6/ 3	5/ 3/ 0	2/ 9/ 4
Time since diagnosis	0–4 weeks	n/a	0–4 weeks

Demographic and clinical characteristics of patients with pulmonary sarcoidosis, pulmonary tuberculosis and healthy volunteers: 14 sarcoidosis patients and 11 healthy volunteers used to source PBMCs for investigation with Kv (Kv study population) and 14 sarcoidosis, 8 healthy volunteers and 15 tuberculosis patients used to source PBMCs for investigation with Kv-specific proteins (Vimentin study population).

The median TNF-α concentration (Fig 1C) was also significantly higher in sarcoidosis PBMCs (2375 pg/mL, IQR 691–6578) compared to healthy volunteer PBMCs stimulated with sKv (42.82 pg/mL, IQR 12.8–107.7, *p* = 0.0003) or sarcoidosis PBMCs stimulated with cKv (10.25 pg/mL, IQR 2.05–19.56, *p* = 0.0001). This pattern was reproducible using biological replicates of sKv and cKv, derived from different spleens (Fig 1B and 1D). There was no difference in secretion of two other measured cytokines (IL-1 and IL-6) in sarcoidosis PBMCs compared to PBMCs from healthy volunteers (data not shown).

### 1D-SDS-PAGE and MS/ MS identifies Kv-specific proteins

**Fig 2:**
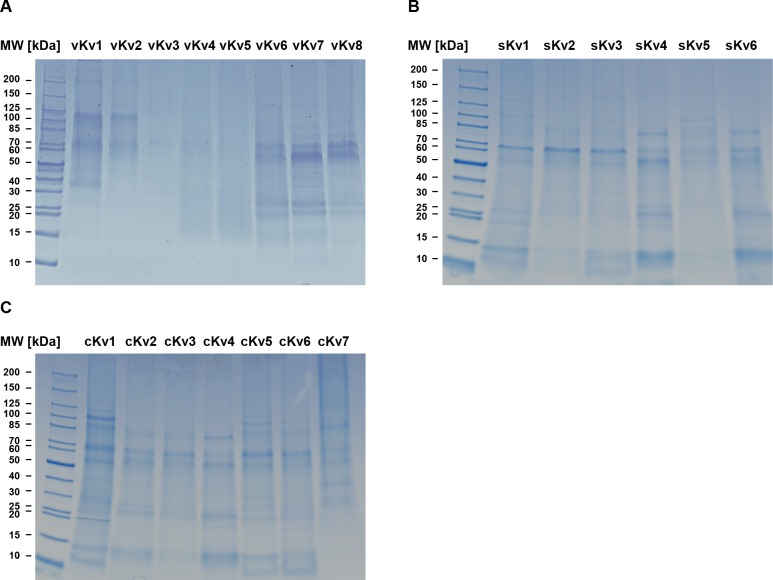
Images of colloidal Coomassie Blue stained 1D-SDS-PAGE gels loaded with biological replicates of historical diagnostically *in vivo* validated Kv (vKv) (Fig 2a), newly created Kv from sarcoidosis spleen (sKv) (Fig 2b) and newly created control Kv from healthy spleen tissue (cKv) (Fig 2c). Each lane was sliced into 10 equal sections. Proteins were subjected to in-gel tryptic digestion and peptides analysed by MS/MS and database searching for protein identification.

Extracted proteins from eight vKv (Fig 2A), six sKv (Fig 2B) and seven cKv (Fig 2C) were separated by 1D-SDS-PAGE and stained with colloidal Coomassie Blue. Lanes were sliced into 10 equal sections and tryptic digests from all sections were subjected to mass spectrometric analysis. A total of 192 proteins were identified from all Kv by searches against the human SwissProt database (version 2012_01) with two independent search algorithms. 48 proteins were identified specific to either sKv and/or vKv i.e. not found in any cKv and these are shown in S1 Table. Seven proteins were present in two or more of the sKv or vKv (Table 2).

**Table 2:** 

Protein	sKv (x/6)	vKv (x/8)	sKv and vKv (x/14)	Highest peptide number	% protein sequence coverage
Tenascin	1	4	5	7	3.6
Fibrinogen gamma chain	0	4	4	7	11.9
Vimentin	1	3	4	5	31.4
78 kDa glucose-regulated protein	1	2	3	4	7.3
Alpha-actinin-4	1	1	2	6	7.7
Tubulin alpha-1B chain	1	1	2	10	9.5
Thioredoxin	0	2	2	36	30.3

A total of 192 proteins were identified from all sarcoidosis and cKv through 1D-SDS-PAGE and MS/MS. Applying stringent search criteria, proteins were considered as accurately identified if they had at least 2 identified peptides and a protein probability 99% /peptide probability 95%. Of these, 48 proteins were specifically found in either sKv or vKv, but not in any cKv. 7 proteins were identified in 2 or more sKv/vKv and are shown in this table e.g. peptides from the protein vimentin were identified in 1 out of 6 sKv samples and 3 out of 8 vKv samples. Therefore the total identification of vimentin peptides across both sKv and cKv was 4 out of 14 samples.

### 2D-DIGE and MS/ MS identifies proteins specific to sarcoidosis spleen

**Fig 3:**
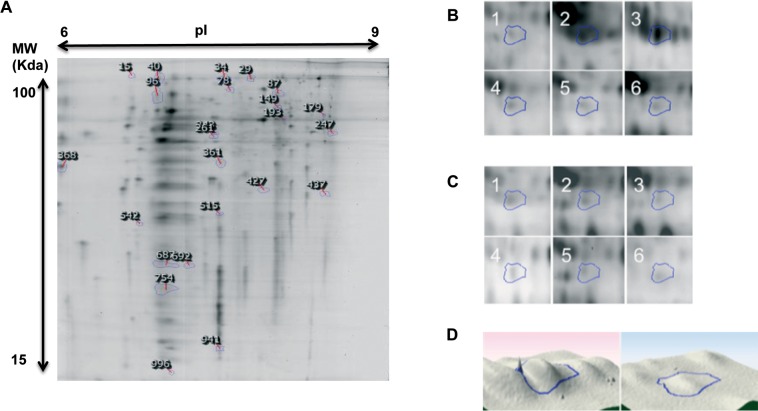
Example of a 2D-DIGE image (pI 6–9) depicting differences in protein abundance between sarcoidosis and healthy control spleen tissue.

The patient cohort used for 1D-SDS-PAGE analysis (sKv and cKv as shown in Fig 2B and 2C) was also subject to 2D-DIGE. 2D-DIGE-gels in the pI range 3–7 resolved 900–1200 protein spots and 250–400 spots in the range 6–9. Protein abundance was assessed by comparison of relative spot volumes from Cy3-labelled sarcoidosis spleen proteins and Cy5-labelled control spleen proteins against a Cy2-labelled internal standard. An example of one 2D-DIGE-gel in pI range 6–9 is shown in Fig 3.

A total of 43 Cy-dye labelled spots identified in sarcoidosis spleen were not seen in normal spleens. Mass spectrometric analyses of these spots resulted in 26 protein identifications (Table 3). A comparative analysis of our two independently generated 1D- and 2D-proteomic datasets identified 3 proteins (vimentin, tubulin and α-actinin-4) that were found in sarcoidosis tissue only and were identified through both 1D-SDS-PAGE and 2D-DIGE.

**Table 3:** 

Protein	Found of increased abundance in x/6 spleens	Highest peptide number	% protein sequence coverage
Actin	2	8	21.1
Vimentin	2	21	43.3
26S proteasome non-ATPase regulatory subunit 9	1	3	13.5
Aldose 1-epimerase	1	3	11.4
Alpha-actinin-4	1	6	7.2
Carbonic anhydrase 2	1	3	10.7
Eukaryotic initiation factor 4A-I	1	2	5.4
Fructose-1,6-bisphosphatase 1	1	11	42.9
Glyoxalase domain-containing protein 4	1	4	14.1
Hemoglobin subunit alpha	1	5	36.6
Hemoglobin subunit delta	1	2	42.9
Ig kappa chain C region	1	4	54.7
Ig lambda-2 chain C regions	1	3	41.5
Keratin, type I cuticular	1	2	5.5
Keratin, type II cuticular	1	6	10.5
Keratin, type II cytoskeletal 1	1	11	14.8
Keratin, type II cytoskeletal 2	1	4	10.5
Keratin, type I cytoskeletal 9	1	4	8.0
Keratin, type I cytoskeletal 10	1	2	3.6
Mannose-1-phosphate guanyltransferase beta 2	1	5	16.9
Peroxiredoxin-2	1	3	15.2
Phosphatidylethanolamine-binding protein 1	1	7	44.4
Proteasome subunit alpha type-7	1	2	7.45
Rab GDP dissociation inhibitor beta	1	12	27.4
Transaldolase	1	5	15.4
Tubulin beta chain	1	7	17.6

Applying stringent search criteria, proteins were considered as accurately identified if they had at least 2 identified peptides and a protein probability 95% /peptide probability 95%. 26 proteins were identified in sarcoidosis spleens only and not in healthy control spleens.

### Stimulation with vimentin induces IFN-γ/ TNF-α secretion from sarcoidosis PBMCs

**Fig 4:**
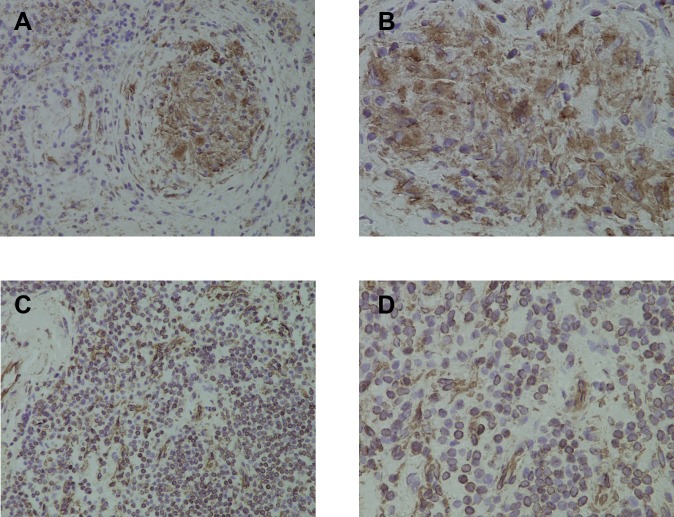
Immunohistochemical staining of spleen tissue shows increased abundance of vimentin in sarcoidosis spleens at 200 x (Fig 4a) and 400 x magnification (Fig 4b) compared to healthy spleen tissue at the same magnifications (Fig 4c and 4d).

Vimentin was identified in through 1D-SDS-PAGE of three historical vKv and one prepared sKv as well as through 2D-DIGE. Immunohistochemical staining of our spleen tissue showed increased abundance of vimentin in sarcoidosis spleens (Fig 4A and 4B) localized particularly to tissue granulomas when compared to healthy spleen (Fig 4C and 4D).

**Fig 5:**
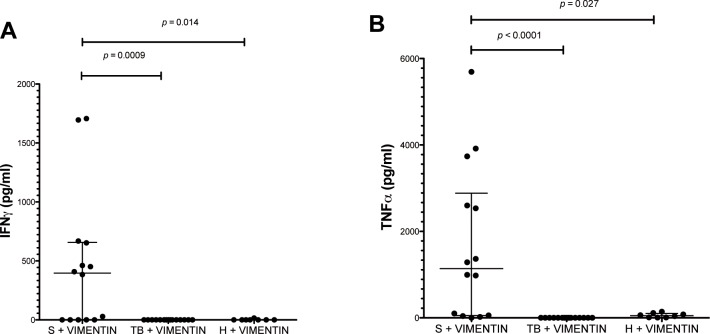
Total IFN-γ (Fig 5a) and TNF-α secretion (Fig 5b) after incubation of PBMCs from patients with sarcoidosis (S), tuberculosis (TB) or from healthy volunteers (H) with pooled recombinant human vimentin. Each dot represents the mean cytokine concentration of one PBMC sample stimulated in duplicate with protein for 36 hrs (after subtraction of the negative control well). Statistical significance between comparisons was determined by Mann-Whitney test.

Multiplex cytokine assays were used to quantify concentrations of secreted IFN-γ, TNF-α, IL-1 and IL-6 from PBMCs after 36 hrs of stimulation with either vimentin, tubulin or α-actinin-4 (see Table 1 for demographic data). After stimulation with vimentin, the median IFN-γ concentration was significantly elevated in the supernatant of sarcoidosis (396.6 pg/mL IQR 0.1–657.1) vs. tuberculosis (0.1 pg/mL IQR 0.1–0.1, *p* = 0.0009) and healthy volunteer PBMCs (0.1 pg/mL IGR 0.1–0.1, *p* = 0.014) (Fig 5A).

TNF-α levels were also significantly elevated in sarcoidosis (1139 pg/mL IQR 51.53–2884) vs. tuberculosis (0.1 pg/mL IQR 0.1–0.1, p<0.0001) and healthy volunteer PBMCs (42.29 pg/mL IQR 7.713–103.4, p = 0.027) (Fig 5B). No difference was observed with IL-1 and IL-6 (data not shown). Tubulin and α-actinin-4, did not elicit any effect in PBMCs from patients with sarcoidosis vs tuberculosis or healthy volunteers (data not shown).

## Discussion

We identified a pro-inflammatory cytokine response in PBMCs from sarcoidosis patients when stimulated with Kveim reagent (Kv). This response was not observed in PBMCs from healthy volunteers stimulated with Kv nor in sarcoidosis PBMCs stimulated with control Kv made from healthy spleen tissue. Using two complementary proteomic approaches we identified 74 proteins specific to sarcoidosis tissue and found that one, vimentin, induced the similar pattern of cytokine secretion from sarcoidosis PBMCs. This response was not seen with vimentin stimulation of PBMCs from healthy volunteers or those with pulmonary tuberculosis.

In the absence of an *in vivo* experimental model, understanding the effect of Kv in granuloma formation is difficult to assess. However, Kv induced granulomas have histologically been detected at 6–24 hrs and resulted in a CD4+ and CD14+ monocyte assembly at 48 hrs after intradermal inoculation [17].

1D-SDS-PAGE coupled to MS/ MS was used to define the Kv proteome and identified 48 proteins specific to sKv and historical vKv. It is noteworthy, that clinical studies have shown that Kv can retain *in vivo* granuloma causing capabilities after lengthy storage e.g. >5 years at 4°C [18]. Comparative 2D-DIGE was then used to identify proteins with higher abundance in sarcoidosis spleen compared to healthy controls.

These two complementary proteomic methods found three proteins in common to both approaches: vimentin, tubulin and alpha-actinin-4. Vimentin is a member of the type III intermediate filaments which take part in cell motility and adhesion, subcellular organization and maintenance of cell shape [19]. It is frequently detected in fibroblasts [20], cells which specifically contribute to advanced, mature granuloma formation [21] and subsequent fibrotic remodeling of tissue [22]. Previous work supports the involvement of several of our identified cytoskeletal proteins in sarcoidosis e.g. tubulin and vimentin have been detected in Schaumann bodies [23] and vimentin was also found within asteroid bodies of sarcoidosis granulomas [24].

The identification of Kv-specific cytoskeletal proteins such as vimentin, tubulin and alpha-actinin-4 might simply reflect increased abundance within granulomatous spleen tissue. However, upon stimulation of sarcoidosis PBMCs with these three candidate proteins, we found that vimentin could induce similarly, a strong and specific IFN-γ and TNF-α secretion as we had observed with Kv itself. A crucial finding is that vimentin did not elicit a pro-inflammatory cytokine response in PBMCs from tuberculosis patients, despite the substantial accumulation of vimentin within tuberculosis granulomas [25].

Rheumatoid arthritis, like sarcoidosis, is a disease where a heightened immune response occurs to a panel of inflammatory stimuli. Intriguingly, a citrullinated form of vimentin is known to be elicit an immune response in rheumatoid arthritis, which correlated with disease activity [26, 27]. Nevertheless, the exact mechanism of the vimentin triggered immune response in rheumatoid arthritis is unknown. It would be intriguing to replicate our experiments and stimulate PBMCs from patients with rheumatoid arthritis and other autoimmune connective tissue disorders (such as SLE or Sjogren’s syndrome) with both vimentin and Kv in an attempt to elict a similar pro-inflammatory cytokine secretion. This would further the hypothesis that there are similarities between those diseases and sarcoidosis in terms of immunopathogenesis.

We could not demonstrate a vimentin-specific response in all sarcoidosis patients. In this respect, it is also important to note that there was no clinical difference (e.g. radiological stage of disease at presentation, age of patient, need for subsequent immunosuppression therapy) between vimentin responders and non-responders.

Our findings support those from recent studies with a specific regard to a role for vimentin in the disease. One group identified a number of vimentin peptides by mass spectrometric sequencing after affinity-purification of HLA-DR molecules (HLA-DRB1*0301) from bronchoalveolar lavage (BAL) cells of sarcoidosis patients. Vimentin-peptides were amongst the identified peptides, suggesting antigenic properties of vimentin in the sarcoidosis lung [28]. Two subsequent analyses revealed differening results with one reporting IFN-γ secretion of PBMCs in responses to the peptide in some sarcoidosis patients expressing the HLA-DRB1*0301 allele [29], but another showing no difference between non-HLA typed sarcoidosis patients and healthy volunteers [30]. We stimulated our PBMCs with whole vimentin protein but individual epitopes of the protein might have differing levels of stimulating capacity in sarcoidosis. We have not HLA typed our subjects but a further analysis of our data revealed that the same patients who exhibited a response to vimentin had also responded to Kv. However, those who responded with IFN-γ secretion were not necessarily the same as those who responded to with TNF-α secretion, suggesting that the PBMC secretion levels of these two pro-inflammatory cytokines were not similar within the same patient.

For the sarcoidosis Kv-reaction, one potential hypothesis is that vimentin merely upregulates cytokine responses from sarcoidosis PBMCs as it is known that other proteins such as mycobacterial-derived antigenic peptides [11] and serum amyloid A [31] can cause a similar response in sarcoidosis. An alternative hypothesis is that vimentin, together with other proteins, may serve as a self-antigen capable of eliciting cytokine responses from sarcoidosis PBMCs, potentially in a CD4+ T-cell mediated fashion. A more recent study has also hypothesised this same role for vimentin in sarcoidosis. This group reported the simultaneous expression of Vα2.3 together with the Vβ22 chain on highly clonal BAL-isolated CD4^+^ T-cells on a cohort of DRB1*03^+^ sarcoidosis patients and molecular modelling indicated a specific T-cell receptor–HLA- DRB1*03^+^- vimentin peptide (Vim_429-443_) interaction [32].

Clinically validated Kv has been historically used to diagnose sarcoidosis with relatively high sensitivity and specificity. Our data, together with that from the most recent publication [32], suggests that the Kv reaction may be an abnormal response to human proteins such as vimentin. This would explain why the reaction has been diagnostic in sarcoidosis patients worldwide, irrespective of the unclear underlying etiology. This also points towards the general concept that such self-antigens may elicit an immune response in sarcoidosis which is common to both the KV reaction and the sarcoidosis granuloma itself.

Interestingly, Kv has also recently been shown to stimulate pro-inflammatory cytokine production of PMBCs from HIV positive patients without sarcoidosis [33]. This unusual finding is difficult to reconcile with our data but it is intriguing that sarcoidosis patients exhibit an abnormal chronic inflammatory immune response, which in some respects has similarities with HIV e.g. the persistent secretion of pro-inflammatory cytokines [34].

Our study has some limitations. Firstly, we have only investigated patients with newly diagnosed pulmonary sarcoidosis. It would be interesting to replicate this work in patients with chronic or quiescent disease as there is some suggestion that Kv skin responses might diminish with time [7]. Although we used a Kv and vimentin concentration similar to that used for PPD stimulation of tuberculosis PBMCs in our previous work [35], we did not perform a dose response curve at different concentrations. Based on our previous work and those of others [17] we also chose a stimulation time similar to that used with stimulation of tuberculosis PBMCs but again, it may be that sarcoidosis PBMCs responding to Kv and vimentin need a different incubation time and this should be investigated further. With regard to our immunological analysis, we have only measured a selection of pro-inflammatory cytokine secretion, as sarcoidosis is known to be a disorder of a heightened Th1 profile with specific elevation of both IFN-γ and TNF-α at the site of disease [5], but it would be important to measure a broader range of cytokine response. Thus, it would be of interest to further unravel the exact nature of the host immune response and to specify the cell type responsible for this observed cytokine secretion from both Kv and vimentin to support the notion that they may be CD4+ T-cells [32]. With regard to our proteomic analysis, gel-based proteomics is only able to cover one portion of the tissue proteome and further techniques such as shotgun proteomics will be needed to further interrogate the Kv proteome for more proteins of interest.

Current diagnosis of sarcoidosis relies on subjective information with patients often being treated with immunosuppressive therapies without a definitive histological diagnosis. We have shown that an *ex vivo* Kv stimulation of sarcoidosis PBMCs elicits a cytokine secretion not seen in controls. Perhaps more importantly, measurement of cytokine secretion in response to one of our identified proteins, vimentin, is shown here to be significantly greater from sarcoidosis PBMCS than those from patients with tuberculosis. Both diseases can present in similar ways and a definitive diagnosis often requires histological sampling. Invasive biopsies are time consuming, expensive and associated with significant morbidity. Our data also represents the first time that self proteins have been identified from within Kveim reagent. A growing number of papers have recently hypothesized that autoimmunity may play an important role in sarcoidosis [36] and we believe that our findings add weight to this hypothesis. If validated in larger cohorts, the cellular responses to vimentin may assist with clinical differentiation between diseases such as sarcoidosis and tuberculosis. Further investigation of Kveim reagent may also result in identification of novel proteins to further understand the pathogenesis of sarcoidosis.

## Supporting Information

S1 Table Full list of the 48 Kv-specific proteins found through 1D-SDS-PAGE and MS/MS. (DOCX)
