# Supplementary material for: Proteomic Analysis of Kveim Reagent Identifies Targets of Cellular Immunity in Sarcoidosis
Source: PLoS One. 2017 Jan 23;12(1):e0170285. doi: 10.1371/journal.pone.0170285 (PMC5256960; doi:10.1371/journal.pone.0170285)
Supplement: S1 Table — (DOCX) [file pone.0170285.s001.docx]

**Supplementary table 1: Full list of 48 proteins identified through 1D-SDS-PAGE in Kv**

| 14-3-3 protein beta/alpha OS=Homo sapiens GN=YWHAB PE=1 SV=3 |
| --- |
| 14-3-3 protein zeta/delta OS=Homo sapiens GN=YWHAZ PE=1 SV=1 |
| 5'-nucleotidase OS=Homo sapiens GN=NT5E PE=1 SV=1 |
| 78 kDa glucose-regulated protein OS=Homo sapiens GN=HSPA5 PE=1 SV=2 |
| ADP-ribosyl cyclase 2 OS=Homo sapiens GN=BST1 PE=1 SV=2 |
| Alpha-actinin-4 OS=Homo sapiens GN=ACTN4 PE=1 SV=2 |
| Alpha-amylase 1 OS=Homo sapiens GN=AMY1A PE=1 SV=2 |
| Annexin A2 OS=Homo sapiens GN=ANXA2 PE=1 SV=2 |
| Annexin A6 OS=Homo sapiens GN=ANXA6 PE=1 SV=3 |
| ATP synthase subunit beta, mitochondrial OS=Homo sapiens GN=ATP5B PE=1 SV=3 |
| ATP synthase subunit O, mitochondrial OS=Homo sapiens GN=ATP5O PE=1 SV=1 |
| ATP-binding cassette sub-family E member 1 OS=Homo sapiens GN=ABCE1 PE=1 SV=1 |
| Collagen alpha-1(V) chain OS=Homo sapiens GN=COL5A1 PE=1 SV=3 |
| Collagen alpha-1(XII) chain OS=Homo sapiens GN=COL12A1 PE=1 SV=2 |
| Cystatin-S OS=Homo sapiens GN=CST4 PE=1 SV=3 |
| Elongation factor Tu, mitochondrial OS=Homo sapiens GN=TUFM PE=1 SV=2 |
| Endoplasmin OS=Homo sapiens GN=HSP90B1 PE=1 SV=1 |
| Erythrocyte band 7 integral membrane protein OS=Homo sapiens GN=STOM PE=1 SV=3 |
| Fibrinogen beta chain OS=Homo sapiens GN=FGB PE=1 SV=2 |
| Fibrinogen gamma chain OS=Homo sapiens GN=FGG PE=1 SV=3 |
| Glial fibrillary acidic protein OS=Homo sapiens GN=GFAP PE=1 SV=1 |
| Glutathione S-transferase kappa 1 OS=Homo sapiens GN=GSTK1 PE=1 SV=3 |
| Guanine nucleotide-binding protein G(k) subunit alpha OS=Homo sapiens GN=GNAI3 PE=1 SV=3 |
| Hornerin OS=Homo sapiens GN=HRNR PE=1 SV=2 |
| Ig heavy chain V-I region EU OS=Homo sapiens PE=1 SV=1 |
| Integrin alpha-M OS=Homo sapiens GN=ITGAM PE=1 SV=2 |
| Isocitrate dehydrogenase [NADP], mitochondrial OS=Homo sapiens GN=IDH2 PE=1 SV=2 |
| Junction plakoglobin OS=Homo sapiens GN=JUP PE=1 SV=3 |
| Keratin, type I cytoskeletal 17 OS=Homo sapiens GN=KRT17 PE=1 SV=2 |
| Keratin-associated protein 2-1 OS=Homo sapiens GN=KRTAP2-1 PE=2 SV=2 |
| Lysozyme C OS=Homo sapiens GN=LYZ PE=1 SV=1 |
| Phosphoglycerate kinase 1 OS=Homo sapiens GN=PGK1 PE=1 SV=3 |
| Platelet factor 4 OS=Homo sapiens GN=PF4 PE=1 SV=2 |
| Prelamin-A/C OS=Homo sapiens GN=LMNA PE=1 SV=1 |
| Proactivator polypeptide OS=Homo sapiens GN=PSAP PE=1 SV=2 |
| Profilin-1 OS=Homo sapiens GN=PFN1 PE=1 SV=2 |
| Prolactin-inducible protein OS=Homo sapiens GN=PIP PE=1 SV=1 |
| Protein disulfide-isomerase A3 OS=Homo sapiens GN=PDIA3 PE=1 SV=4 |
| Protein S100-A11 OS=Homo sapiens GN=S100A11 PE=1 SV=2 |
| Protein S100-A6 OS=Homo sapiens GN=S100A6 PE=1 SV=1 |
| Stabilin-1 OS=Homo sapiens GN=STAB1 PE=1 SV=3 |
| Tenascin OS=Homo sapiens GN=TNC PE=1 SV=3 |
| Thioredoxin OS=Homo sapiens GN=TXN PE=1 SV=3 |
| Trinucleotide repeat-containing gene 18 protein OS=Homo sapiens GN=TNRC18 PE=1 SV=3 |
| Tubulin alpha-1B chain OS=Homo sapiens GN=TUBA1B PE=1 SV=1 |
| Tubulin beta chain OS=Homo sapiens GN=TUBB PE=1 SV=2 |
| Vimentin OS=Homo sapiens GN=VIM PE=1 SV=4 |
| Voltage-dependent anion-selective channel protein 3 OS=Homo sapiens GN=VDAC3 PE=1 SV=1 |
